# Advances in Analytical Strategies to Study Cultural Heritage Samples—2nd Edition

**DOI:** 10.3390/molecules30193952

**Published:** 2025-10-01

**Authors:** Maria Luisa Astolfi, Maria Pia Sammartino

**Affiliations:** 1 Department of Chemistry, Sapienza University of Rome, Piazzale Aldo Moro 5, 00185 Rome, Italy; mariapia.sammartino@uniroma1.it; 2 Research Center for Applied Sciences to the Safeguard of Environment and Cultural Heritage (CIABC), Sapienza University of Rome, Piazzale Aldo Moro 5, 00185 Rome, Italy

## 1. Introduction

The study of cultural heritage samples represents a unique intersection of science, history, and art, where the careful application of analytical techniques can uncover invaluable information about materials, manufacturing processes, degradation phenomena, and conservation strategies. In recent years, advances in analytical chemistry, spectroscopy, microscopy, and multi-modal imaging have revolutionized the approach to heritage science, allowing researchers to obtain detailed compositional and structural information while minimizing sample destruction [1]. These methods are particularly crucial for irreplaceable artifacts, historical documents, wall paintings, textiles, and archaeological finds, where traditional sampling strategies may be limited or impossible.

Recent trends in heritage science have emphasized non-invasive and minimally invasive techniques, reflecting both ethical considerations and technological progress [1,2]. For instance, vibrational spectroscopies such as Fourier transform infrared (FTIR) and Raman spectroscopy, in combination with chemometric data analysis, provide precise information on organic and inorganic components without compromising the artifact’s integrity [3,4]. Similarly, hyperspectral imaging and portable X-ray fluorescence (pXRF) allow in situ chemical mapping of materials in complex cultural contexts [5]. The application of mass spectrometry, including gas chromatography–mass spectrometry (GC–MS) and ambient mass spectrometry techniques, has further enabled the detailed characterization of organic binders, pigments, and volatile compounds [6,7,8,9,10]. Such technological integration has also been complemented by nanomaterial applications, where engineered nanoparticles and functionalized hydrogels offer innovative strategies for conservation, cleaning, and protective coatings [11,12].

The multidisciplinary nature of heritage research has encouraged collaborations among chemists, materials scientists, conservators, art historians, and archaeologists. This collaborative approach not only improves the reliability of analytical results but also ensures that scientific findings are translated into practical conservation strategies. In addition, computational modeling and data-driven approaches, including multivariate statistical analysis and machine learning algorithms, have become increasingly valuable for interpreting complex datasets and predicting long-term behavior of materials under various environmental conditions [13].

Recent studies have highlighted the importance of developing comprehensive analytical workflows that integrate multiple techniques, allowing for cross-validation of results and deeper insights into the chemical and physical transformations occurring in cultural heritage objects [14,15]. Examples include the identification of metal soap formation in oil paints, the detection of early synthetic dyes in textiles, and the non-invasive characterization of historic print techniques [16,17,18,19,20]. The continuous refinement of these methodologies enhances both preventive conservation strategies and forensic studies, providing robust evidence for provenance, authenticity, and degradation assessment [21,22,23,24].

Moreover, the growing awareness of environmental and anthropogenic impacts on heritage materials has reinforced the need for continuous monitoring and tailored interventions [25,26]. From airborne pollutants affecting paper and textiles to light-induced photodegradation of pigments, understanding the mechanisms of deterioration is essential for designing effective conservation protocols [27,28,29,30,31]. By integrating chemical, physical, and environmental data, contemporary heritage science enables informed decision-making for the preservation of artifacts across centuries.

Overall, the contributions to the second edition of the Special Issue “Advances in Analytical Strategies to Study Cultural Heritage Samples” exemplify the diversity and sophistication of current research, illustrating how modern analytical strategies can both expand scientific understanding and support practical conservation efforts.

## 2. An Overview of Published Articles

The second edition of the Special Issue comprises nine contributions, which collectively highlight state-of-the-art in heritage science analytical strategies. Doménech-Carbó et al. (contribution 1) represented a compelling integration of heritage conservation methodologies with forensic science approaches, addressing a highly sensitive historical context: the mass graves of the Francoism era in Spain (1939–1956). The authors employed a comprehensive multi-analytical strategy to examine personal objects recovered from these sites, including clothing, metal objects, and personal effects, aiming to both preserve the material integrity of the items and extract critical forensic information. Non-destructive techniques such as XRF, FTIR, and optical microscopy were combined with targeted sampling strategies to provide a detailed chemical and physical characterization of the artifacts. The study not only revealed the composition and degradation patterns of the materials but also offered insights into the manufacturing techniques and potential provenance of the objects. By bridging conservation science and forensic analysis, this work underscores the potential of analytical chemistry to support both historical reconstruction and ethical handling of sensitive cultural heritage. Furthermore, it highlights the importance of developing protocols that minimize sample handling while maximizing the information obtainable from fragile and invaluable items, setting a precedent for future studies in similar socio-historical contexts. This contribution exemplifies how interdisciplinary approaches can enhance the understanding of both material culture and historical events, combining methodological rigor with societal relevance.

Chiodini et al. (contribution 2) highlighted the application of modern analytical chemistry techniques to historical book conservation. The authors investigated volatile organic compounds (VOCs) emitted by two rare books, aiming to understand degradation processes and chemical interactions within paper and ink components. Using gas chromatography–mass spectrometry (GC–MS) and headspace analysis, they identified specific VOC profiles associated with oxidation, hydrolysis, and microbial activity. The work demonstrates how VOC monitoring can serve as an early diagnostic tool to detect degradation before visible damage occurs, providing crucial information for preventive conservation strategies. This non-invasive approach ensures the preservation of the integrity of the historical texts while offering actionable insights for long-term storage and environmental control.

Botti et al. (contribution 3) explored innovative nanocomposite hydrogels as cleaning and protective agents for paper-based artworks. The study focused on poly(acrylic acid)/TiO_2_ formulations, which combine mechanical cleaning efficacy with photocatalytic antimicrobial properties. The hydrogels were thoroughly characterized for their rheological behavior, swelling capacity, and interaction with paper fibers. Application tests demonstrated effective removal of surface contaminants without compromising the paper structure. This research underscores the potential of nanotechnology for cultural heritage conservation, offering environmentally friendly solutions that enhance both cleaning efficiency and long-term protection.

Labate et al. (contribution 4) employed a combination of non-invasive techniques—including XRF, UV–visible diffuse reflectance spectrophotometry with optical fibres (FORS), and portable X-ray diffraction (XRD)—to investigate the chemical transformations responsible for the blackening of originally blue paint layers. The multi-analytical approach allowed the identification of pigment composition, degradation products, and environmental factors contributing to discoloration. The study provides valuable insights into both the deterioration mechanisms and suitable conservation strategies, demonstrating how integrated non-destructive analysis can support informed decision-making in artwork preservation. Further exploring paint systems, the study by Poli et al. (contribution 5) provided initial insights into metal soap formation in alkyd-based paints using FTIR spectroscopy. The authors investigated the early stages of this common degradation phenomenon, monitoring the chemical interactions between metal ions and fatty acid components. The results offer proof-of-concept data that enhance understanding of degradation pathways and underscore the importance of early detection for preventive conservation. Such findings can guide conservators in selecting suitable environmental and storage conditions to mitigate metal soap-related deterioration. Freixas-Jambert et al. (contribution 6) focused on bromoil photographic prints, which present unique conservation challenges due to their complex organic and inorganic components. Using external reflection FTIR spectroscopy as a non-invasive technique, the study successfully identified key binder and pigment constituents, providing insights into historical photographic processes and degradation mechanisms. This work underscores the value of minimally destructive analytical methods for the characterization, study, and preservation of sensitive photographic heritage.

Textile heritage was explored by Forleo et al. (contribution 7), who applied ATR-FTIR spectroscopy combined with multivariate curve resolution–alternating least squares (MCR-ALS) algorithms to profile early synthetic dyes on historic woolen samples. This approach allowed the authors to deconvolute complex overlapping spectral signals, identifying the presence of early synthetic dyes while preserving the integrity of the delicate fibers. By providing detailed chemical information on historical dyeing techniques, this study contributes to understanding the material composition, degradation pathways, and conservation needs of textile artifacts.

The insights gained from such advanced spectroscopic and chemometric analyses resonate with complementary strategies demonstrated in other contributions of the Special Issue. De Caro et al. (contribution 8) explored the functionalization of artwork packaging using Ag-doped TiO_2_ and ZnO nanoparticles, providing innovative solutions for the preventive conservation and protection of cultural heritage materials. Similarly, Nádvorníková et al. (contribution 9) employed GC–MS combined with principal component analysis to differentiate lipid binders in wall paintings, illustrating how sophisticated analytical workflows can resolve complex organic mixtures and inform appropriate conservation strategies. Together, these studies exemplify the integration of non-destructive and minimally invasive analytical techniques with chemometrics and material functionalization approaches, highlighting the breadth of modern heritage science from chemical characterization to preventive conservation.

## 3. Conclusions

The second edition of the Special Issue highlights the continued evolution of analytical strategies in heritage science, emphasizing minimally invasive and multi-modal approaches. The studies presented demonstrate that integrating advanced spectroscopy, mass spectrometry, imaging, and nanomaterials can provide comprehensive insights into both the composition and degradation of cultural artifacts. Furthermore, these contributions underscore the importance of multidisciplinary collaboration, combining expertise in chemistry, materials science, conservation, and art history to achieve meaningful results.

The editorial emphasizes that future research should continue to develop accessible, robust, and non-destructive methods while considering sustainability and environmental impacts. As analytical technologies advance, researchers are better equipped to address long-standing conservation challenges, improve preventive care, and inform policy decisions regarding cultural heritage management. The achievements of this Special Issue exemplify the scientific and practical value of contemporary analytical strategies, and they provide a roadmap for future investigations in the field.
